# Sertoli Cells Modulate Testicular Vascular Network Development, Structure, and Function to Influence Circulating Testosterone Concentrations in Adult Male Mice

**DOI:** 10.1210/en.2016-1156

**Published:** 2016-05-04

**Authors:** Diane Rebourcet, Junxi Wu, Lyndsey Cruickshanks, Sarah E. Smith, Laura Milne, Anuruddika Fernando, Robert J. Wallace, Calum D. Gray, Patrick W. F. Hadoke, Rod T. Mitchell, Peter J. O'Shaughnessy, Lee B. Smith

**Affiliations:** Medical Research Council Centre for Reproductive Health (D.R., J.W., L.C., S.E.S., L.M., A.F., R.T.M., L.B.S.), University/BHF Centre for Cardiovascular Science (J.W., P.W.F.H.), and Clinical Research Imaging Centre (C.D.G.), University of Edinburgh, The Queen's Medical Research Institute, Edinburgh EH16 4TJ, United Kingdom; Department of Orthopaedics (R.J.W.), University of Edinburgh, Edinburgh Eh16 4SB, United Kingdom; and Institute of Biodiversity, Animal Health, and Comparative Medicine (P.J.O.), University of Glasgow, Garscube Campus, Glasgow G61 1QH, United Kingdom

## Abstract

The testicular vasculature forms a complex network, providing oxygenation, micronutrients, and waste clearance from the testis. The vasculature is also instrumental to testis function because it is both the route by which gonadotropins are delivered to the testis and by which T is transported away to target organs. Whether Sertoli cells play a role in regulating the testicular vasculature in postnatal life has never been unequivocally demonstrated. In this study we used models of acute Sertoli cell ablation and acute germ cell ablation to address whether Sertoli cells actively influence vascular structure and function in the adult testis. Our findings suggest that Sertoli cells play a key role in supporting the structure of the testicular vasculature. Ablating Sertoli cells (and germ cells) or germ cells alone results in a similar reduction in testis size, yet only the specific loss of Sertoli cells leads to a reduction in total intratesticular vascular volume, the number of vascular branches, and the numbers of small microvessels; loss of germ cells alone has no effect on the testicular vasculature. These perturbations to the testicular vasculature leads to a reduction in fluid exchange between the vasculature and testicular interstitium, which reduces gonadotropin-stimulated circulating T concentrations, indicative of reduced Leydig cell stimulation and/or reduced secretion of T into the vasculature. These findings describe a new paradigm by which the transport of hormones and other factors into and out of the testis may be influenced by Sertoli cells and highlights these cells as potential targets for enhancing this endocrine relationship.

The testicular vasculature forms a complex capillary bed, interdigitating between the seminiferous tubules to provide oxygenation, delivery of micronutrients, and clearance of waste from the testis. Impairment of the testicular vasculature, for example, the reduction in venous drainage observed in cases of varicocele, causes intratesticular hypoxia and germ cell apoptosis (1). The vasculature is also instrumental to the endocrine function of the testis because it is the route by which pituitary gonadotropins are delivered to the testis to support T production and spermatogenesis (2). Conversely, alongside the lymphatic system, the vascular system is important for transport of T to other body systems; a reduced testis and vascular volume is associated with a reduction in circulating T concentrations (3).

Our understanding of the mechanisms by which the testis controls local vascular function in adulthood is extremely limited. There is some evidence that testicular mast cells can influence vascular blood flow through release of 5-hydroxytryptamine (4), but perhaps the most well-studied factor influencing testicular vascular function is T. T is a well-established regulator of testicular vasomotion (rhythmical contraction and relaxation of blood vessels, independent of heartbeat) (5, 6) via direct T-mediated activation of the androgen receptor in smooth muscle cells of the testicular vasculature (7).

Speculation that Sertoli cells may influence the testicular vasculature is supported by some indirect evidence (5) and in vitro studies (8), but confirmation of a direct role for Sertoli cells in the regulation of the testicular vasculature in vivo has never been demonstrated unequivocally. Recently we developed a unique model system that uses diphtheria toxin to specifically and acutely ablate Sertoli cells from the testis (9, 10). This model has revealed several important, yet previously unknown, roles that Sertoli cells play in neonatal and adult life (reviewed in reference 11).

In this study we used models of acute Sertoli cell ablation and acute germ cell ablation, to address whether Sertoli cells actively influence vascular function in the adult testis. Our findings suggest that Sertoli cells play a key role in supporting the structure of the testicular vasculature and describe a new paradigm by which the transport of hormones and other factors into and out of the testis can be influenced by Sertoli cells and highlights these cells as potential targets for enhancing this endocrine relationship.

## Materials and Methods

### Ethics statement

Mice were housed and bred under standard conditions of care. Experiments passed local ethical review and were conducted with licensed permission under the UK Animal Scientific Procedures Act (1986) (Home Office license number PPL 60/4200).

### Mouse tissue collection

Animals with selective Sertoli cell ablation (9, 10) or germ cell ablation (12) were generated and tissue collected, as previously described.

### Testis dissociation and xenografting

Testis dissociation into a single cell suspension, pelleting in Matrigel, and subcutaneous xenografting under the back skin of castrated male CD1 nude mice were completed as previously described (13). Xenografts were retrieved 4 weeks later, weighed, and fixed in Bouin's solution for 2 hours.

### Resin perfusion

Mice were culled using a terminal dose of sodium pentobarbital (150 mg/kg, ip). Anterograde perfusion fixation of the vasculature was achieved via the left ventricle. Heparinized PBS (heparin, 20 U/mL) was infused at 6 mL/min for 2 minutes. Low-viscosity resin (10 mL; Microfil MV-122; Flow Tech Inc) was prepared according to the manufacturer's instructions and then infused via the left ventricle. Tissues were fixed in paraformaldehyde.

### Optical projection tomography (OPT) and microcomputed tomography (μCT) scanning of resin cast testes

Resin cast testes were processed for OPT as previously described (14) with a final isotropic voxel size of 8.07 × 8.07 × 8.07 μm^3^. For μCT, samples were placed in a Skyscan 1172 μCT (Bruker) as previously described (15) and scanned with the following parameters: voxel resolution 3.44 μm, voltage 41 kV, current 240 μA, exposure 1767 msec, and a 0.5-mm aluminum filter. The reconstructed data were processed by CTvox software (Skyscan) and quantified by CTAn software (Skyscan). During the scanning and reconstruction process, the identity of the samples was blinded.

### Vessel density quantification and image analysis procedure

Vessel density was quantified using ImagePro plus 7.0 on tiled images in which vessels were identified by vascular marker Von Willebrand factor (VWF; red). Images were binarized, with the vessels in white and the background in black. The vessel density was reported relative to the xenograft area. A vessel analysis was performed on three-dimensional (3D) OPT and μCT volumes in the software package (Fiji) (16) using the Analyze Skeleton plugin (17).

### RNA isolation and real-time PCR

Real-time PCR and analysis was carried out as previously described and normalized against an external standard (luciferase) (10). The primers used are described in Supplemental Table 1.

### Histological analysis

Hematoxylin and eosin staining and immunofluorescent localization was carried out as previously described (10). The primary antibodies used were CD31 (Abcam Ltd; ab28364), VWF (Dako; A0082), cleaved caspase-3 (Cell Signaling (NEB); number 9661), DDX4 (DEAD-box helicase 4) (Abcam Ltd; ab13840), and SOX9 (Sex Determining Region Y-Box 9) (Millipore; ab5535).

### Vasculature-testis fluid exchange

Animals with selective Sertoli cell or germ cell ablation and their littermate controls were iv injected with 200 μL Evans Blue dye (0.5% in PBS) 30 minutes prior to culling. Tissue was processed and extravasation quantified as previously described (18).

### Cell preparation

Testicular cell suspensions were prepared from three to five adult animals per group as described (19) and incubated with recombinant human LH (10^−9^ M) (LH; MerckSerono) (20). To normalize the data for the Leydig cell number, results were determined relative to the stable expression of *Hsd3b1* transcript levels measured in nonincubated cell aliquots (21–23). Each experiment was performed in triplicate on three separate occasions, and the overall data from the three experiments are reported.

### T quantification

T levels were determined as previously described (10). One cohort of animals received a single ip injection of 20 IU human chorionic gonadotropin (hCG) (Pregnyl; Organon) 16 hours prior to tissue collection.

### Statistical analysis and image handling

Data (mean ± SEM) were analyzed using a Student *t* test or an ANOVA as appropriate) using GraphPad Prism (version 6; GraphPad Software Inc) or Minitab (version 15 Minitab Inc). When required, data were normalized by box-cox or log transformation. Images were compiled as previously described (9).

## Results

### Vascular analysis after induced cell ablation

Amh-Cre^+/+^;iDTR^+/+^ mice were generated, and the specificity and efficacy of Sertoli cell ablation demonstrated, as previously described (9–11). Briefly, an injection of 100 ng of diphtheria toxin (DTX) induces apoptosis of the Sertoli cell population within 1 day, whereas an injection of a vehicle control has no effect. In this study, day 50 adult males were injected with 100 ng DTX and tissue collected 7 or 30 days later. Because the loss of Sertoli cells also leads to a complete loss of the germ cell population within 30 days (9), we replicated all analyses in a cohort of mice treated with busulfan, which specifically removes only the germ cell population (12). This cohort not only provided a comparator between impacts of germ cell loss vs Sertoli cell loss, but because both treatment groups exhibit the same reduction in testis volume, this also acted as a control for any overall change in organ size, which is known to influence the total volume of intraorgan vascularization in a predictable manner (24).

**Figure 1.**
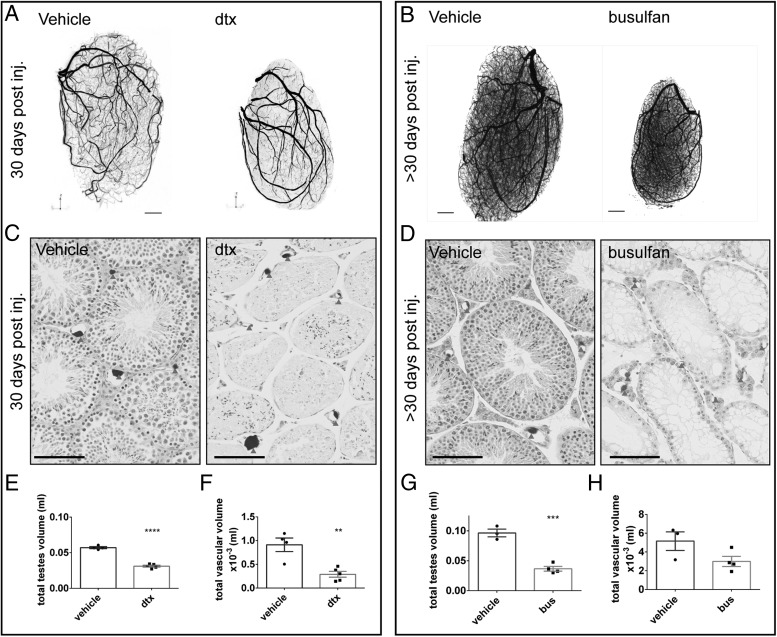
Vascular analysis after Sertoli cell-induced cell ablation and germ cell-induced cell ablation. Adult testes from Amh-Cre;iDTR mice treated with DTX and collected 30 days after Sertoli cell ablation (A) and germ cell-ablated animal (B) were resin casted, scanned by μCT or OPT, and 3D images reconstructed. Scale bar, 0.1 cm. C and D, The contralateral testes were processed for histology and the testicular gross histology revealed the presence of functional vessels (arrowheads) Scale bar, 100 μm. Total testis volume was significantly reduced (E) 30 days after Sertoli cell ablation or germ cell ablation and total vascular volume was reduced (G) after Sertoli cell ablation (F) but remained unchanged after germ cell ablation (H). (*t* test, n = 3–6). **, *P* < .05; ***, *P* < .001; ****, *P* < .0001. bus, injected with busulfan; DTX, injected with toxin; post inj., post injection.

Mice were perfused with a lead radioopaque resin prior to tissue collection. Testes were scanned by μCT and OPT to provide high-resolution 3D reconstructions (Figure 1, A and B, and Supplemental Figure 1A). Hematoxylin and eosin staining of testicular tissue sections from contralateral testes confirmed Sertoli cell ablation in DTX-treated testes and germ cell ablation in the busulfan-treated testis and confirmed that at both 7 days or 30 days after the ablation, the gross morphology of functional blood vessels was intact and that all blood vessels contained resin (Figure 1, C and D, arrowheads, and Supplemental Figure 1B). Importantly, despite the loss of Sertoli and/or germ cells, seminiferous tubules, although reduced in diameter in both groups, remained structurally intact in all samples (ie, retaining a defined basement membrane and peritubular cells, which continue to separate the tubules from the interstitium), ensuring that gross testicular architecture was maintained.

### Total vascular volume is reduced after Sertoli cell, but not germ cell, ablation

Quantitative analysis of OPT and μCT images showed that testicular volume was significantly reduced in the 7- (*P* < .01) and 30-day (*P* < .0001) Sertoli cell-ablated testes and 30-day germ cell-ablated testes (Figure 1, E–G, and Supplemental Figure 1C), consistent with previous observations (9, 12). Total vascular volume was unchanged in 7-day Sertoli cell-ablated testes and 30-day germ cell-ablated testes but was significantly decreased 30 days after Sertoli cell ablation *P* < .001, (Figure 1, F–H, and Supplemental Figure 1D), suggesting Sertoli cells, but not germ cells, play an important role in the long-term support of the testicular vascular network.

To determine how the vascular volume becomes reduced by 30 days after Sertoli cell ablation, we examined the testes 1, 3, 7, and 30 days after Sertoli cell ablation for evidence of increased cell apoptosis (cleaved caspase-3 immunolocalization) in vessels (immunostained with endothelial marker VWF). Although Sertoli cell and germ cell apoptosis was observed within the seminiferous tubules at 3 and 7 days after Sertoli cell ablation, no evidence of endothelial cell apoptosis was found, suggesting any vascular apoptosis occurs between 7 days and 30 days after Sertoli cell ablation (Supplemental Figure 2) and that the reduction in vascular volume observed in response to Sertoli cell loss therefore occurs over an extended period of several weeks.

### Sertoli cells increase testicular microvessel density in a xenograft model

**Figure 2.**
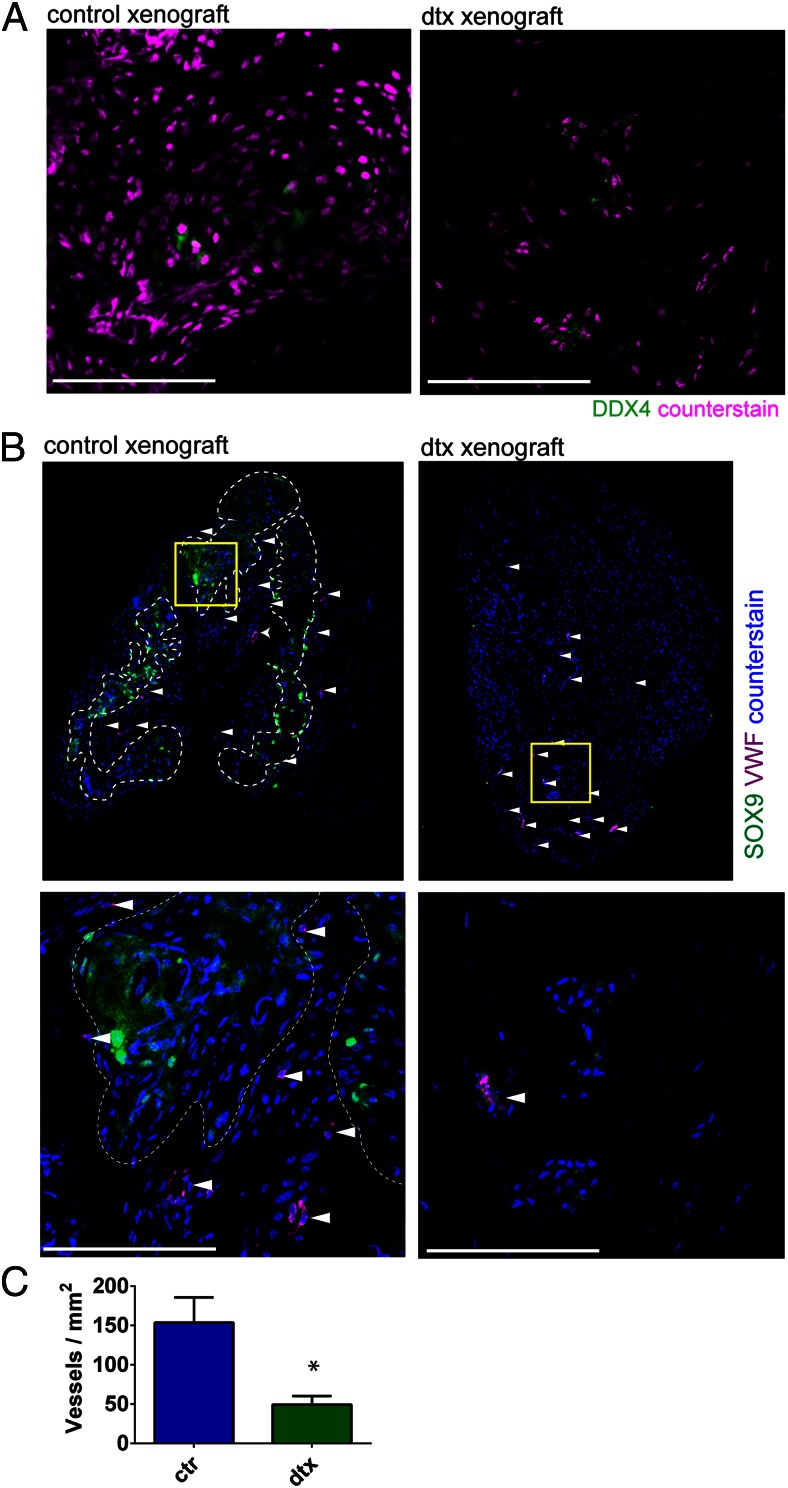
Sertoli cells increase testicular microvessel density in a xenograft model. Control and Sertoli cell-ablated testes were dissociated to a single-cell suspension, pelleted in matrigel, and xenografted under the skin of nude mice. A, Germ cell marker DDX4 showed few germ cells are present in the xenografts. Scale bar, 100 μm. B, Sertoli cell marker SOX9 and vascular marker VWF coimmunolocalization showed that Sertoli cells are required for reformation of seminiferous tubules (delimited by the dotted lines) and confirmed the presence of microvessels within the graft, respectively (white arrows). Scale bar, 100 μm. C, Microvessel density per xenograft area was quantified histologically using the specific endothelial marker VWF. This revealed a significant decrease in microvessel density in xenografts lacking Sertoli cells (*t* test, n = 3–4). *, *P* < .05). ctr, control; dtx, injected with DTX.

To further assess the relationship between Sertoli cells and vascular development and retention, we used a well-developed model of testis dissociation and xenografting (10, 25, 26). Isolated control testes and Sertoli cell-ablated testes were made into single-cell suspensions 7 days after Sertoli cell ablation (to control for any difference in testicular architecture), pelleted in Matrigel, and xenografted under the skin of nude mice; grafts were collected 4 weeks later. Consistent with our own and other previous published studies (10, 13, 26), germ cell survival was minimal in all xenografts (Figure 2A). This negated any potential impact of germ cells on vascularization and permitted us to directly assess the impact of Sertoli cell presence or absence on microvessel formation. Microvessels were apparent (immunostaining for VWF) in xenografts both with or without Sertoli cells (Figure 2B), but quantification of the microvessel density demonstrated that the presence of Sertoli cells significantly increased the numbers of microvessels within the graft (Figure 2C). This suggests that Sertoli cells positively influence the numbers of microvessels in their local environment.

### Sertoli cell ablation, but not germ cell ablation, reduces vascular-testis fluid exchange

**Figure 3.**
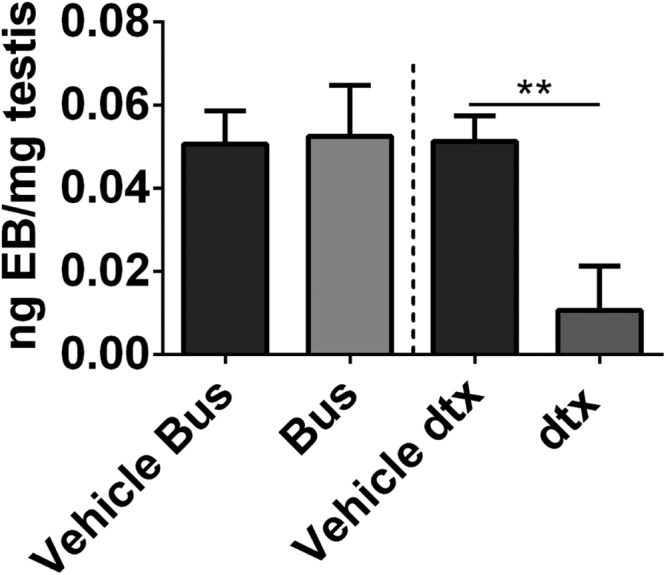
Sertoli cell ablation, but not germ cell ablation, reduces the vascular-testis fluid exchange. Vascular-testis fluid exchange was measured by quantification of Evans blue extravasation. The OD read at 620 nm was converted to nanograms of dye extravasated per milligram of tissue. Extravasation after busulfan treatment was comparable between vehicle and germ cell-depleted testes. However, in the absence of Sertoli cells, the vascular extravasation was significantly decreased (*t* test, n = 3–6). **, *P* < .01. Bus, injected with busulfan; Vehicle Bus, vehicle for the Bus group; vehicle dtx, vehicle for the DTX group; dtx, injected with DTX.

We next assayed for functional impacts by examining fluid exchange between the vascular system and the testis. We quantified fluid flow into the testis indirectly using the well-established ability of Evans blue dye to bind albumin. The amount of extravasation of albumin-bound dye from blood vessels into the interstitium directly correlates with the total vascular permeability of a tissue (18). Because LH is known to increase vascular-tissue fluid exchange (28), we first validated this approach on a cohort of animals treated with the alternative LHCGR (luteinizing hormone/choriogonadotropin receptor) agonist, hCG, 16 hours prior to collection. As expected, fluid flow into the testis was significantly increased in hCG-treated animals compared with unstimulated controls, confirming that the method will detect changes in fluid exchange (Supplemental Figure 3A). To examine the role of the Sertoli cells in regulating this, the fluid exchange was measured in Sertoli cell-ablated mice (>30 d after Sertoli cell ablation; without hCG stimulation) and was shown to be significantly reduced (*P* < .01) (Figure 3). In contrast, vascular permeability was unaffected after germ cell ablation (>30 d after germ cell ablation; without hCG stimulation) despite a similar reduction in testis weight (Figure 3). Heart and lung control tissues showed no difference in fluid exchange, confirming that the observed reduction in fluid flow in Sertoli cell-ablated testis of DTX-treated mice, is a direct result of the removal of Sertoli cells from the testis (Supplemental Figure 3, B and C).

### Normal vascular branching in the adult testis is Sertoli cell dependent

**Figure 4.**
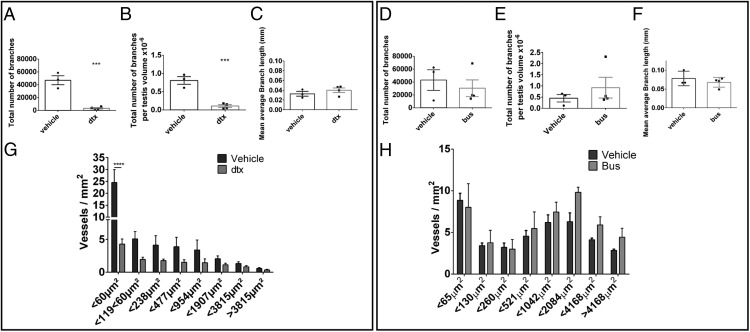
Normal vascular branching and number of microvessels in the adult testis are Sertoli cell dependent. The total number of vascular branches (A) and the total number of vascular branches per testis volume (B) were significantly decreased 30 days after Sertoli cell ablation, but the mean average branch length (C) did not change (*t* test, n = 3–6). ***, *P* < .001. All three parameters remained unchanged after germ cell ablation (D–F). The number of microvessels with a diameter less than 60 μm^2^ was significantly reduced in Sertoli cell-ablated testes when compared with untreated controls (G) (two way ANOVA with a Tukey post hoc test, n = 3–6). ****, *P* < .0001. However, normal vessel distribution was retained after germ cell ablation when compared with untreated controls (H). Bus, busulfan.

To establish the anatomical mechanism by which fluid exchange is impaired, we characterized the branching of the intratesticular vascular network. The total number of vascular branches (junctions between vessels), the number of branches corrected for total testis volume, and the mean average branch length were all unchanged 7 days after Sertoli cell ablation (Supplemental Figure 4, A–C). However, at 30 days after the Sertoli cell ablation, although there was again no change in branch length, we did observe a significant reduction in the total number of vascular branches, which was independent of total testis volume (Figure 4, A–C). Importantly, we did not observe a similar reduction in total branch number in testes 30 days after germ cell ablation (Supplemental Figure 4, D–F), which strongly indicates that the reduction in total branch numbers is a response to the absence of Sertoli cells.

### Microvessel number in the adult testis is Sertoli cell dependent

**Figure 5.**
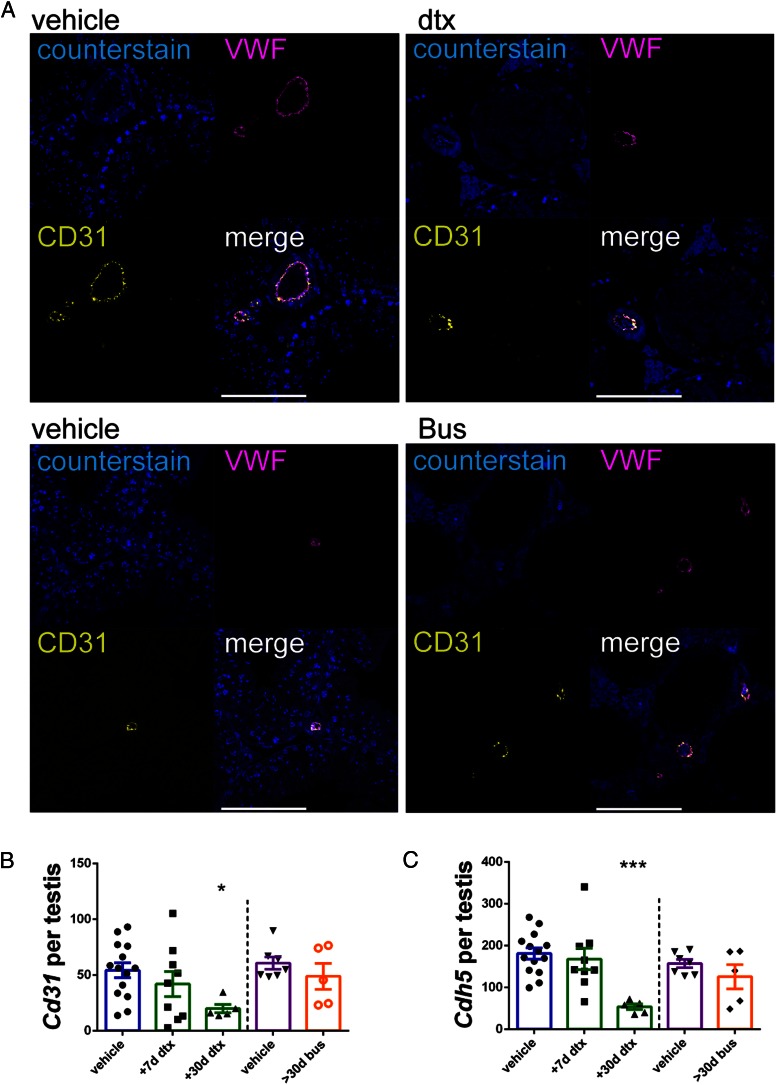
Endothelial markers expression is reduced after Sertoli cell, but not germ cell, ablation. Established endothelial cell markers VWF and CD31 were confirmed to be restricted to endothelial cells of the testicular vasculature (A). Scale bar, 100 μm. Expression of both endothelial markers (B) *Cd31* and (C) *Cdh5* did not vary 7 days after Sertoli cell ablation or after germ cell ablation but did exhibit a significant reduction 30 days after Sertoli cell ablation (one way ANOVA, n = 5–9 on DTX samples and *t* test on bus samples). *, *P* < .05; ***, *P* < .001). Bus, injected with busulfan; dtx, injected with DTX; post inj., after injection.

To assess the impact of Sertoli cell ablation on vessel size distribution, vessel size was determined on central transverse sections derived from each 3D reconstructed image. At 7 days after Sertoli cell ablation, vessel size distribution did not differ between treated and control animals (Supplemental Figure 4D). At 30 days after Sertoli cell ablation, however, although all larger vessels showed no difference between groups, we did observe a significant reduction in microvessels with a cross-sectional area less than 60 μm^2^ (Figure 4G). This was not seen in testes from the 30-day postgerm cell ablation, which showed no change in vessel number of any size (Supplemental Figure 4H). The reduction in microvessel numbers observed 30 days after the Sertoli cell ablation was supported by a significant reduction in the gene expression of the confirmed endothelial cell-specific markers CD31 (Figure 5A) and CDH5 only in this group (Figure 5, B and C). Together these results indicate that the retention of microvessels with a diameter less than 60 μm^2^ in the adult testis is also Sertoli cell dependent.

### Reduced microvessel number explains reduced fluid exchange after Sertoli cell ablation

To complete this analysis, we then compared the reduction in microvessel number to the observed reduction in fluid flow into the testicular interstitium (Supplemental Figure 5). Correcting for the reduction in microvessel number normalizes the difference in fluid flow observed between Sertoli cell-ablated and control testes, suggesting that the reduction in numbers of microvessels explains most, if not all, of the difference in fluid flow into the testis observed between the two groups (Supplemental Figure 5).

### Reduced fluid exchange impairs the stimulation of Leydig cell T production

**Figure 6.**
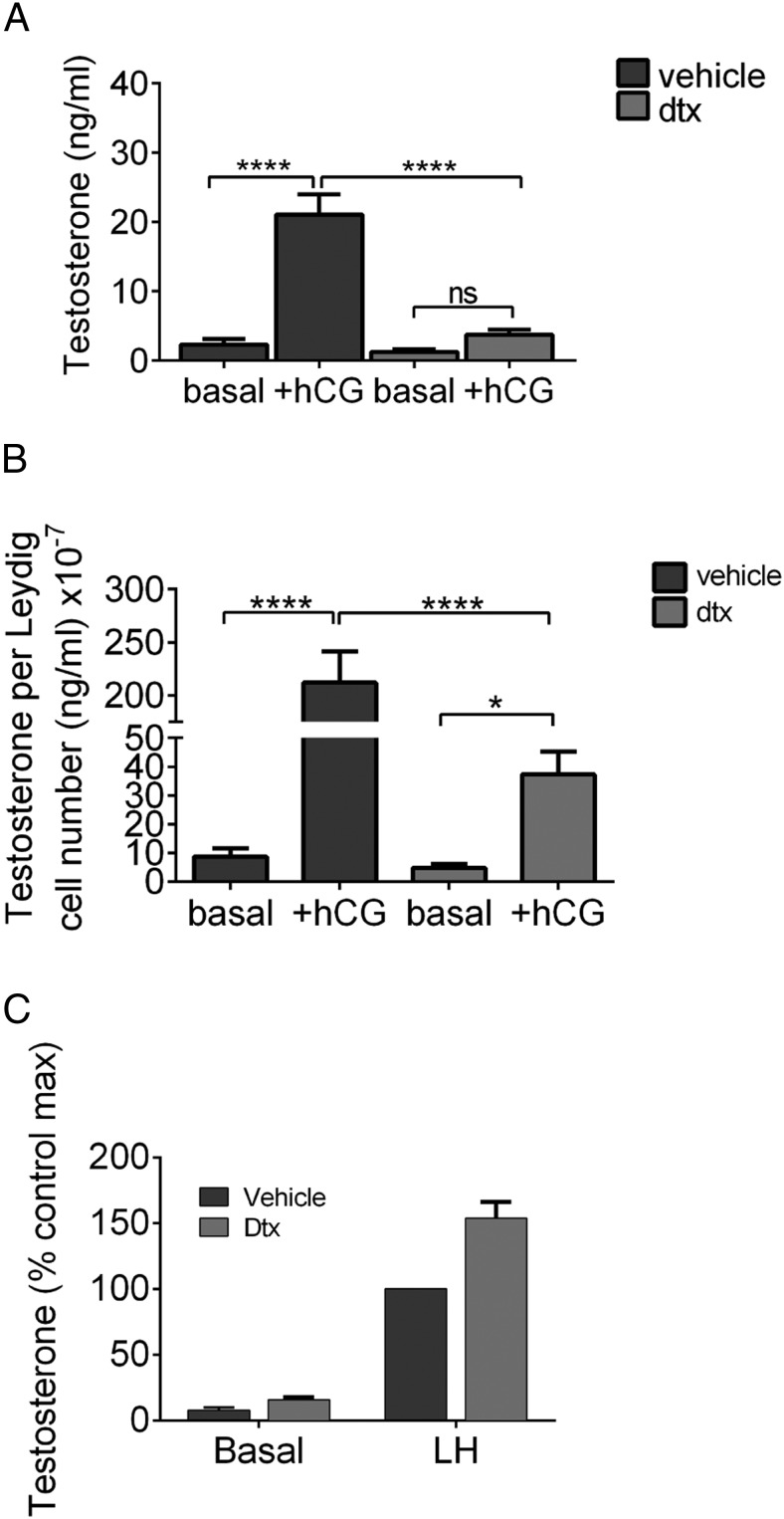
Reduced fluid exchange impairs stimulation of Leydig cell T production. Leydig cell response to in vivo gonadotropin (hCG) stimulation was assessed 30 days after Sertoli cell ablation. Both total circulating T (A) (two way ANOVA, n = 5–20, ****, *P* < .0001) and circulating T relative to total Leydig cell number (B) (two way ANOVA, n = 5–20, *, *P* < .05, ****, *P* < .0001) were significantly reduced. C, Isolated testicular cells were incubated under basal conditions or in the presence of LH (10^−9^ M) and T production analyzed. Results show the accumulated data (mean ± SEM) from three experiments normalized to the maximum control T level in each experiment. Each experiment was in triplicate and data were analyzed by a three-way ANOVA (experiment, LH and DTX were factors). This analysis showed a significant overall stimulatory effect of DTX, and post hoc testing showed that the effect of DTX was significant in each individual experiment.

The significant reduction in fluid exchange could affect T production by the Leydig cells through reducing LH stimulation of these cells. To address this possibility, we injected control or Sertoli cell-ablated animals (30 d after the DTX injection) with hCG (another LHCGR agonist) and then measured circulating T concentrations 16 hours later. Sertoli cell ablation caused a significant reduction in hCG-stimulated T concentrations (Figure 6A), which was not completely explained by correcting for the reduction in Leydig cell numbers observed in this model (9) (Figure 6B). This raises the possibility that Leydig cells in the Sertoli cell-ablated mice are being understimulated by the circulating hCG due to compromised fluid flow. Alternatively, it is possible that Leydig cells in Sertoli cell-ablated testes are less active and do not respond as well to trophic stimulation or that reduced fluid exchange reduces secretion of T into the vasculature. To examine this further, we isolated Leydig cells from control and Sertoli cell-ablated testes and stimulated them directly in vitro with LH (Figure 6C). Isolated Leydig cells from both groups responded to LH treatment. Leydig cells from the Sertoli cell-ablated testis in fact showed a hyperactive response to LH.

**Table 1.** Antibody Table

Peptide/Protein Target	Antigen Sequence (if Known)	Name of Antibody	Manufacturer, Catalog Number, and/or Name of Individual Providing the Antibody	Species Raised (Monoclonal or Polyclonal)	Dilution Used
CD31	Synthetic peptide corresponding to C terminus of mouse CD31	CD31	Abcam Ltd, ab28364	Rabbit polyclonal	1:100
VWF		VWF	Dako, A0082	Rabbit polyclonal	1:1500
DDX4	Synthetic peptide conjugated to KLH derived from within residues 700 to the C terminus of human DDX4/MVH	DDX4	Abcam Ltd, ab13840	Rabbit polyclonal	1:400
Cleaved caspase-3	Antibody detects endogenous levels of the large fragment (17/19 kDa) of activated caspase-3 resulting from cleavage adjacent to Asp175	Cleaved caspase-3	Cell Signaling (NEB), number 9661	Rabbit polyclonal	1:500
SOX9		SOX9	Millipore, ab5535	Rabbit polyclonal	1:4000

## Discussion

The definition of an endocrine gland is that it secretes its product into the vascular system, leading to a subsequent action at target organs. In the testis, which both receives endocrine stimulation (primarily, but not exclusively, by gonadotropins) and secretes androgens into the circulation in response to this stimulation, the development, integrity, and function of the vascular network is essential. Despite the importance of the vasculature, the factors that control vascular integrity and function in the adult testis have remained largely unknown. In contrast, the role of Sertoli cells in promoting many key aspects of testis development and function has expanded in recent times (9–11). In this study, we now demonstrate that Sertoli cells are also essential for normal regulation of the testicular vasculature. Specific ablation of Sertoli cells in vivo leads to a reduction in total intratesticular vascular volume, the number of vascular branches, and the numbers of small microvessels. Absence of Sertoli cells in a testis xenograft model also reduces microvessel numbers within the graft. Together these observations support an active role for Sertoli cells in the development and maintenance of the testicular microvessel network.

Perturbations to the vascular network after Sertoli cell ablation leads to a reduced capacity for fluid exchange, as indicated by a reduction in gonadotropin-stimulated circulating T concentrations, despite the high steroidogenic potential of Leydig cells in the Sertoli cell-ablated testis. Our evidence suggests that the failure to respond normally to hCG when stimulated in vivo (ie, failure to increase circulating T concentrations) is likely to be due to a reduction in the numbers of microvessels, the reduced numbers of endothelial cells (important for transcytosis of LH/hCG into the testis (29, 30), and, by definition, a reduced surface area for fluid exchange (hCG in and T out). The dual role of LH/hCG in terms of both increasing vascular fluid exchange in the testis (Supplemental Figure 3) (28, 31) and also directly stimulating T production remain underexplored. Our data suggest that the ability to enhance fluid exchange may be an important role because hCG treatments fail to produce the expected response in hyperactively primed Leydig cells present in the Sertoli cell ablated testis; this requires further study. Together these data suggest that Sertoli cell support for the retention of testicular microvessels in the adult testis forms a hitherto unrecognized yet key component of the endocrine system of the testis. Importantly, our studies also show that the germ cells have little influence over the testicular vasculature.

The mechanisms by which the Sertoli cells influence the testicular vascular structure and function are not clear. The most likely mechanisms are through specific Sertoli cell-derived factors or through secondary paracrine signals arising from the Leydig cells or peritubular cells. There is good evidence that the Leydig cells regulate testicular vasomotion through the secretion of T (6, 32), which acts through androgen receptor on vascular smooth muscle cells (33). In addition, ablation of the Leydig cells suppresses the proliferation of endothelial cells and active remodeling of the testicular vasculature, whereas the transplantation of interstitial cell grafts (but not isolated tubules) leads to development of a prominent vascular network around the graft (34). Ablation of the Sertoli cells leads to a 50% loss of the Leydig cell population by 30 days (9), so changes in the testicular vasculature may be a result of lost interaction between these cell types. There is also evidence, however, that the Sertoli cells can secrete factors that stimulate endothelial cell proliferation (27) and formation of capillary-like structures in vitro (8), so further study is required to identify the underlying mechanism.

In conclusion, the findings presented here describe a new paradigm by which the transport of hormones and other factors into and out of the testis is influenced by Sertoli cells and highlights Sertoli cells as potential targets for enhancing this endocrine relationship.
